# Personality inventory for DSM-5 brief form(PID-5-BF) in Chinese students and patients: evaluating the five-factor model and a culturally informed six-factor model

**DOI:** 10.1186/s12888-021-03080-x

**Published:** 2021-02-17

**Authors:** Panwen Zhang, Zirong Ouyang, Shulin Fang, Jiayue He, Lejia Fan, Xingwei Luo, Jianghua Zhang, Yan Xiong, Fusheng Luo, Xiaosheng Wang, Shuqiao Yao, Xiang Wang

**Affiliations:** 1 Medical Psychological Center, The Second Xiangya Hospital of Central South University, Changsha, Hunan China; 2 Hunan Biological and Electromechanical Polytechnic, Changsha, Hunan China; 3 Institute of Mental Health, The Second Xiangya Hospital of Central South University, Changsha, Hunan China; 4 Student Affairs Department, Central South University, Changsha, Hunan China; 5 Student Affairs Department, Central South University of Forestry and Technology, Changsha, Hunan China; 6 Department of Human Anatomy and Neurobiology, Xiangya School of Medicine, Central South University, Changsha, Hunan China

**Keywords:** Personality inventory for DSM-5-brief form (PID-5-BF), Factor structure, Reliability, Validity, Measurement invariance, Personality diagnostic questionnaire-4+ (PDQ-4+)

## Abstract

**Background:**

The Personality Inventory for DSM-5 Brief Form (PID-5-BF) is a 25-item measuring tool evaluating maladaptive personality traits for the diagnosis of personality disorders(PDs). As a promising scale, its impressive psychometric properties have been verified in some countries, however, there have been no studies about the utility of the PID-5-BF in Chinese settings. The current study aimed to explore the maladaptive personality factor model which was culturally adapted to China and to examine psychometric properties of the PID-5-BF among Chinese undergraduate students and clinical patients.

**Methods:**

Seven thousand one hundred fifty-five undergraduate students and 451 clinical patients completed the Chinese version of the PID-5-BF. Two hundered twenty-eight students were chosen randomly for test-retest reliability at a 4-week interval. Exploratory factor analysis (EFA) and confirmatory factor analysis (CFA) were conducted to discover the most suitable factor structure in China, measurement invariance(MI), internal consistency, and external validity were also calculated.

**Results:**

The theoretical five-factor model was acceptable, but the exploratory six-factor model was more applicable in both samples (Undergraduate sample: CFI = 0.905, TLI = 0.888, RMSEA = 0.044, SRMR = 0.039; Clinical sample: CFI = 0.904, TLI = 0.886, RMSEA = 0.047, SRMR = 0.060). In the Chinese six-factor model, the Negative Affect domain was divided into two factors and the new factor was named “Interpersonal Relationships”, which was in line with the Big-Six Personality model in Chinese. Measurement invariance across non-clinical and clinical sample was established (configural, weak, strong MI, and partial strict MI). Aside from acceptable internal consistency (Undergraduate sample: alpha = 0.84, MIC = 0.21; Clinical sample: alpha = 0.86, MIC = 0.19) and test-retest reliability(0.73), the correlation between the 25-item PID-5-BF and the 220-item PID-5 was significant(*p* < 0.01). The six PDs measured by Personality diagnostic questionnaire-4+ (PDQ-4+) were associated with and predicted by expected domains of PID-5-BF.

**Conclusions:**

Both the theoretical five-factor model and the exploratory six-factor model of the PID-5-BF were acceptable to the Chinese population. The five-factor model could allow for comparison and integration with other work on the original theoretical model. However, the Chinese six-factor structure may be more culturally informed in East Asian settings. In sum, the PID-5-BF is a convenient and useful screening tool for personality disorders.

## Background

Personality disorders (PDs) are common psychiatric conditions that are disruptive to everyday functioning. Because PDs are often misdiagnosed or missed entirely, reliable and valid clinical diagnostic tools for PDs are needed [1]. In light of the numerous weaknesses of the traditional PD taxonomic diagnostic system, including a high comorbidity rate, arbitrary cutoff scores, and substantial heterogeneity within PDs in Section III of the DSM-5 [2], the APA (2013) proposed an Alternative Model of Personality Disorder (AMPD) diagnosis for six PDs (antisocial, avoidant, borderline, narcissistic, obsessive-compulsive and schizotypal), wherein 25 maladaptive personality trait facets are organized into five domains (Criterion B) after measuring personality functioning (Criterion A) [3]. The AMPD provides theoretical trait domains across six specified PDs, thus converting PD diagnosis from a categorical to a dimensional scheme.

The Personality Inventory for DSM-5 (PID-5) is a 220-item self-report measurement that was developed specifically to evaluate hierarchically organized personality traits in accordance with the AMPD. The reliability and validity of the PID-5 have been confirmed in multiple studies, which have yielded internal consistency values above 0.8 for most domains [4–7]. The five broad domains affirmed in prior studies [8, 9] have been described as comparable to maladaptive variants of the Big-Five Model [10], which would be expected given that the development of the associated dimensional model of personality pathology was informed by the normal personality taxonomy [11]. Although the PID-5 has satisfactory psychometric properties, its utility is limited due to its having a large number of items, and thus its taking substantial time to complete [12]. With the aim of screening for PDs quickly and accurately, the PID-5 Brief Form (PID-5-BF) was developed by extracting core items from the five domains (Negative Affect, Detachment, Antagonism, Disinhibition, and Psychoticism) of the PID-5 [13]. Correlation coefficients between the five PID-5-BF domains and the original PID-5 domains have been reported to be very good, with Bach et al. (2016) reporting a mean correlation coefficient value of 0.90 [14], and Debast et al. (2017) reporting correlation coefficients in the range of 0.81–0.87 [15].

PID-5-BF results provide an overall assessment of degree of personality maladjustment and point to potential PDs [16]. The PID-5-BF has been shown to be reliable and valid in a number of countries in Europe [14–18], North America [19–21], and Asia [22]. In all but two cases, internal consistency coefficients above 0.8 were obtained; internal consistency coefficients for Belgium [15] and Denmark [14] were between 0.66 and 0.78. The PID-5-BF has been shown to have satisfactory discriminant validity (effect size of mean domain level = 0.46, *p* < .05) between individuals with and without PDs [14]. In terms of criterion validity, each PD measured by Personality Diagnostic Questionnaire-4(PDQ-4) was associated with and predicted by theoretical PID-5-BF domains. Regarding internalizing and externalizing criteria, the PID-5-BF Negative Affect domain sub-score is a robust predictor of Inventory for Depression and Anxiety Symptoms-2 scores. Meanwhile, the PID-5-BF Disinhibition and Antagonism domain sub-scores are specifically predictive of Externalizing Spectrum Inventory scale scores. Somewhat unexpectedly, PID-5-BF Psychoticism domain subscores were found to be significantly predictive of scores for the aforementioned three scales [20]. PID-5-BF domain sub-scores and total scores correlated significantly with Personality Assessment Screener scores [21].

Although the PID-5 domains (Negative Affect, Detachment, Antagonism, Disinhibition, and Psychoticism) have been understood as putative maladaptive variants of Big-Five Model dimensions (Neuroticism, Extraversion, Agreeableness, Conscientiousness, Openness) respectively, the appropriateness of the Big-Five Model differs across cultures. For example, the Big-Five Model was found not well-fitted in India [23], the Philippines [24], Korea [25], and Japan [26]. In consideration of cultural dissociations of personality constructs, it is also of interest to note that the replicability of the Openness domain of the NEO Personality Inventory was also found to be poor in Asian countries in a cross-cultural study that included 24 cultures [27]. In addition, a six-factor personality model has been discovered to be more suitable than the five-factor structure in Chinese settings [28–34]. As one dimension of Big Six personality, Interpersonal Relationship was raised based on the Chinese context [28, 35], which reflected the humanistic ethic spirit of Chinese culture. Song et al. [31] developed the Chinese personality assessment inventory(CPAI) to measure the six-factor model, and in Cheung’s research, joint factor analysis on CPAI and NEO-PI-R confirmed the unique factor (Interpersonal Relatedness) loaded only by CPAI and none of the NEO-PI-R facets, which encompasses the traditional Chinese concepts of relationship orientation (Ren Qing; i.e. reciprocity of favors, affections, etc.), Harmony (e.g. lack of conflict, balance), and Face (as in “saving face” or reputation) [36, 37]. Similarly, the five-factor model of the PID-5-BF did not achieve an adequate fit in a study of Filipino college students [22]. Given that the five-factor model of the PID-5-BF has been supported principally in studies with Western samples, it is likely that cultural differences may underlie differing factor structure findings in the literature. Accordingly, the most suitable factor structure of the PID-5-BF in Chinese respondents remains to be clarified. In addition, Measurement invariance (MI) studies of the PID-5-BF should be conducted to determine whether factor structure differs between populations constituted by individuals of different cultures [38, 39]. Although MI of the PID-5 has been examined across cultures, sexes, and across clinical and non-clinical samples [40–42], the MI of the PID-5-BF is unknown.

The current study was the first to examine the psychometric properties of the Chinese version of the PID-5-BF. The aims of this study were threefold. First, we set out to determine the most suitable factor structure of the PID-5-BF in a Chinese population sample. Second, we assessed MI of the Chinese version of PID-5-BF across undergraduate students and clinical patient samples, which is important for the generalization of the PID-5-BF in both research and clinical settings. Third, to investigate criterion-related validity, we analyzed how well PID-5-BF scores correlate with scores obtained on the 220-item PID-5 and PDQ-4+, also conducted multiple regression analysis.

## Methods

### Participants

Seven thousand nine hundred eighty-five university students were recruited from two Chinese universities in Hunan Province from 2015 to 2017. After omitting subjects with missing data values, we retained a final undergraduate sample of 7155 subjects (3713 male, 51.9%, and 3436 female, 48.0%) with a mean age of 18.26 [standard deviation (SD), 1.33; range 18–23] years.

The clinical sample was collected from 2018 to 2020, including 451 outpatients (205 men, 45.5%, and 246 women, 54.5%), with a mean age of 24.62 (SD, 8.49; range, 18–57) years, who had been referred to the psychological clinic in hospital for assessment and treatment. Each patient was diagnosed based on the Structured Clinical Interview for DSM-IV system by two experienced psychiatrists (WX, LXW). The distribution of diagnoses in the clinical sample was as follows: PD, 43.2%; major depressive disorder, 22.6%; anxiety disorder, 4.9%; bipolar disorder, 7.1%; obsessive-compulsive disorder, 4.9%; schizophrenia, 3.1%; and other mental disorders (substance use disorders, somatic symptom disorder, eating disorder, Post-traumatic stress disorder), 14.2%.

All study procedures were approved by the Ethics Committee of Second Xiangya Hospital, Central South University. All participants signed informed consent.

### Instruments

#### Chinese PID-5 and PID-5-BF

**Fig. 1:**
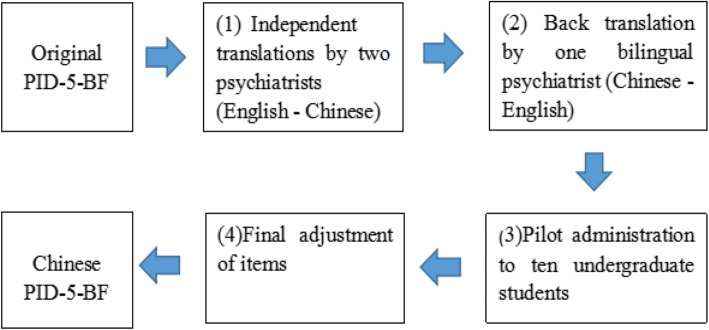
Flowchart of procedures for translation of the Personality Inventory for the DSM-5-Brief Form (PID-5-BF)

The full-length PID-5 [43] is a 220-item self-report scale developed in the USA to index 25 lower-order trait facets (Cronbach’s α, 0.72–0.96) organized into five higher-order trait domains of personality pathology (Cronbach’s α, 0.84–0.96) [43]. We invited two psychologists to translate the PID-5 scale from American English into Chinese; and then it was translated back into English by a bilingual teacher, with repeated revisions to ensure translation accuracy. We evaluated the scale in 10 undergraduate students, after the final adjustment of items, this version of PID-5 was set down (concrete steps were shown in Fig. 1). The PID-5-BF [44] was developed by extracting 25 items from the original PID-5, representing 21 of the 25 trait facets (facets not included: Restricted Affectivity, Rigid Perfectionism, Submissiveness, and Suspiciousness). Items are rated on a 0–3 Likert-type scale, with higher scores representing greater dysfunction. Each of the five higher-order domains is represented by five items (Negative Affect: Items 8, 9, 10, 11, and 15; Detachment: Items 4, 13, 14, 16, and 18; Antagonism: Items 17, 19, 20, 22, and 25; Disinhibition: Items 1, 2, 3, 5, and 6; and Psychoticism: Items 7, 12, 21, 23, and 24).

#### PDQ-4+

The PDQ-4+ [45] is a self-report PD assessment scale based on the DSM-4. Items are answered as “yes” (scored as 1) or “no” (scored as 0). The Chinese version of the PDQ-4+ [46] used in the current study contains 107 items constituting 12 PD-type sub-scales. The PDQ-4+ has been used reliably in PD studies in China [47, 48], with Cronbach’s α values ranging from 0.49 (Passive-Aggressive) to 0.72 (Depressive).

### Model testing procedures

In the undergraduate sample, 3985 students finished the 220-item PID-5, and 7155 students completed the PID-5-BF and PDQ-4+. For the clinical sample, we obtained 451 valid PID-5-BF, 395 valid PID-5, and 231 valid PDQ-4+ questionnaires. For evaluation of test-retest reliability, 228 undergraduate sample participants (93 male, 135 female) were chosen randomly for a PID-5-BF re-test taken 4 weeks after the initial test. Construct validity of the Chinese PID-5-BF was assessed with exploratory factor analysis (EFA) and confirmatory factor analysis (CFA) after randomly (roughly) halving the undergraduate sample randomly into an EFA subsample (*N* = 3633) and a CFA subsample (*N* = 3522). CFA was conducted to test the theoretical model suggested being the best fitting model in the EFA.

MI tests across the population included four nested models. Model 1 (configural invariance) tested the factor structure of latent variables across our two population samples with all parameters freely estimated. Model 2 (weak invariance) was based on the configural results with factor loadings equalized across groups. Next, Model 3 (strong invariance) had the consistency of variable intercepts; achieving this model indicates that latent factor scores have the same meaning across groups, and thus that group comparisons are tenable. Model 4 (strict invariance) requires equalized error variance on the basis of the previous three models [49], which is rarely achieved [50].

### Data analysis

Data analysis was performed in IBM SPSS Statistics 23.0 and Mplus 7.4. To examine construct validity, we first conducted EFA to identify the most suitable factor model of the PID-5-BF. Oblique rotation was used to allow correlation among factors. Each item with a factor loading ≥0.3 was accepted as a factor component [51]. For EFA and CFA, model fit indices applied included the comparative fit index (CFI), the Tucker-Lewis index (TLI), the standard root mean square residual (SRMR), and the root mean square error of approximation (RMSEA) with a 90% confidence interval (CI). Acceptable fit values were: CFI ≥ 0.90, TLI ≥ 0.90, SRMR ≤0.08, and RMSEA ≤0.08 [52]. Model modifications were made on the basis of item correlations and MI index values.

MI was estimated based on three indices, namely △CFI, △TLI, and △RMSEA, wherein △ represents the difference between two adjacent models. Invariance was verified when △CFI and △TLI were ≤ 0.01 and △RMSEA was < 0.015 [53]. In cases where these criteria were not met, the largest-value modification indices were selected to determine the parameters of which item(s) should be released to be free across groups iteratively until the △CFI was ≤0.01, demonstrating potential partial invariance [40].

Internal consistency was determined by calculating Cronbach’s α and mean inter-item correlation (MIC) values. Cronbach’s α > 0.8 and > 0.9 signified acceptable and good reliability, respectively; a MIC > 0.15 was considered acceptable [54]. Test-retest reliability was estimated by calculating a Pearson’s correlation coefficient (r). To examine criterion validity, Pearson’s r index values were also calculated between the PID-5-BF and the original PID-5, as well as between the PID-5-BF and the PDQ-4+ (representing the six DSM-4 PDs retained in DSM-5, Section III). Pearson r values > 0.30 and > 0.50 indicated medium and large effect sizes, respectively [55]. Finally, multiple regression analysis was performed to evaluate multivariate models in which the PID-5-BF domains predicted PDQ-4+ scores using a step-by-step method. △R^2^ was used to measure the effect size of each variate, higher value represents a stronger predictive effect; the variance inflation factor (VIF) was calculated in order to assess collinearity [56], < 5 represents non-collinearity among variables.

## Results

### Descriptive statistics

The mean (SD) PID-5-BF total score obtained for the full university student sample (*N* = 7155) was 16.4 (8.20), with females having a significantly greater (t = − 6.80, *p* < 0.01) mean score, at 17.10 (7.96), than males, at 15.79 (8.32). The mean (SD) PID-5-BF total score obtained for the clinical sample (*N* = 451) was 29.78 (10.70), with females again showing a significantly greater (t = − 4.37, *p* < 0.01) mean score, at 31.75 (10.98), than males, at 27.41 (9.86). The mean obtained for the clinical sample was significantly greater (t = 26.04, *p* < 0.01) than that obtained for undergraduate student sample. The mean (SD) PID-5-BF total score obtained for the sub-sample used in the EFA, at 16.29 (8.24) (*N* = 3633), was statistically similar (t = − 1.34, *p* = 0.18) to that obtained for the sub-sample used in the CFA, at 16.55 (8.11) (*N* = 3522).

### EFA and CFA

EFA supported an exploratory five-factor model and an exploratory six-factor model. The factor designations of the items are compared across these two exploratory models and Krueger et al.’s (2013) previously reported five-factor model [44] in Table 1. In our exploratory five-factor model, five items (8, 9, 11, 15, and 20) did not load into any factor; item 10 was the only item to reach a 0.3 loading weight in the Negative Affect domain, and it loaded with item 19, which had belonged to the Antagonism factor in the previously published model. Our exploratory six-factor model had higher fit indices (CFI = 0.969, TLI = 0.944) than the exploratory five-factor model (CFI = 0.952, TLI = 0.922), with fewer items failing to load on any factor (items 8 and 11). A new factor named Interpersonal Relationships was added to the original five factors. The factor loading of each item in the exploratory six-factor model are reported in Table 2.

**Table 1:** Item comparison among three models

Model	Items associated with each factor, F1–6					
	F1	F2	F3	F4	F5	F6
Theoretical five-factor	8, 9, 10, 11, 15	4, 13, 14, 16, 18	17, 19, 20, 22, 25	1, 2, 3, 5, 6	7, 12, 21, 23, 24	–
Exploratory five-factor	–	4, 13, 14, 16, 18	17, 22, 25	1, 2, 3, 5, 6	7, 12, 21, 23, 24	10, 19
Exploratory six-factor	9, 15	4, 13, 14, 16, 18	17, 20, 22, 25	1, 2, 3, 5, 6	7, 12, 21, 23, 24	10, 19

**Table 2:** Factor loading for the exploratory six-factor model (*N* = 3633)

Item	Factor					
	NA	IR	De	An	Ps	Di
9. I get emotional easily, often for very little reason.	**0.70**	0.05	0.00	−0.07	0.12	0.02
15. I get irritated easily by all sorts of things.	**0.50**	0.00	0.23	0.10	−0.03	0.02
10. I fear being alone in life more than anything else.	0.01	**0.61**	−0.02	−0.01	− 0.02	0.02
19. I crave attention.	0.06	**0.47**	−0.11	0.09	0.01	−0.00
4. I often feel like nothing I do really matters.	−0.05	0.06	**0.44**	0.01	0.01	0.17
13. I steer clear of romantic relationships.	0.04	−0.11	**0.36**	−0.10	0.21	−0.01
14. I’m not interested in making friends.	0.06	−0.12	**0.58**	0.07	0.01	0.00
16. I don’t like to get too close to people.	0.04	−0.08	**0.43**	0.03	0.21	−0.10
18. I rarely get enthusiastic about anything.	−0.02	0.03	**0.59**	−0.01	0.12	0.02
17. It’s no big deal if I hurt other peoples’ feelings.	0.04	0.01	0.28	**0.43**	−0.12	0.07
20. I often have to deal with people who are less important than me.	0.08	0.14	0.06	**0.30**	0.23	−0.06
22. I use people to get what I want.	0.01	0.02	0.05	**0.61**	0.03	0.10
25. It is easy for me to take advantage of others.	−0.05	0.00	−0.02	**0.65**	0.22	−0.02
7. My thoughts often don’t make sense to others.	0.04	−0.06	0.07	0.01	**0.45**	0.17
12. I have seen things that weren’t really there.	0.00	0.00	0.06	0.16	**0.42**	−0.01
21. I often have thoughts that make sense to me but that other people say are strange.	−0.02	− 0.05	−0.08	0.14	**0.61**	0.08
23. I often “zone out” and then suddenly come to and realize that a lot of time has passed.	0.04	0.14	0.03	−0.14	**0.52**	0.10
24. Things around me often feel unreal, or more real than usual.	−0.00	0.10	0.05	0.02	**0.63**	−0.03
1. People would describe me as reckless.	0.15	−0.05	−0.15	0.06	−0.03	**0.55**
2. I feel like I act totally on impulse.	0.15	−0.01	−0.01	0.01	0.02	**0.56**
3. Even though I know better, I can’t stop making rash decisions.	0.07	0.07	0.05	−0.01	0.20	**0.37**
5. Others see me as irresponsible.	−0.02	−0.00	0.23	0.07	0.07	**0.35**
6. I’m not good at planning ahead.	−0.07	0.12	0.17	−0.14	0.00	**0.46**
8. I worry about almost everything.	0.17	0.19	0.22	0.14	0.04	0.06
11. I get stuck on one way of doing things, even when it’s clear it won’t work.	0.06	0.16	0.24	0.05	0.12	0.21

*NA* Negative Affect, *IR* Interpersonal Relationships, *De* Detachment, *An* Antagonism, *Ps* Psychoticism, *Di* Disinhibition

Bold represents the largest factor loading in each item as well as > 0.30

We chose to pursue an analysis of our exploratory six-factor model because it had better model fit indices and fewer items that failed to load than our exploratory five-factor model and because of the significant differences in the Negative Affect domain between our exploratory five-factor model and the theoretical five-factor model. On the whole, both the theoretical five-factor model and exploratory six-factor model were acceptable. However, as shown in Table 3, CFA results showed that the six-factor model has achieved better indices when compared with the theoretical five-factor model, which indicated that the six-factor model would be more culturally-adapted in Chinese settings. As the theoretical five-factor model failed to meet the precondition of measurement invariance, we chose the six-factor model to conduct further analysis.

**Table 3:** Goodness of fit index values for the compared models

Model	CFI	TLI	SRMR	RMSEA	RMSEA 90%CI	
					LO90	HI90
Normal sample (*N* = 3522)						
TFF	0.887	0.872	0.044	0.046	0.044	0.048
ESF	0.905	0.888	0.039	0.044	0.042	0.046
Clinical sample (*N* = 451)						
TFF	0.868	0.848	0.061	0.053	0.048	0.059
ESF	0.904	0.886	0.060	0.047	0.041	0.054

*TFF* theoretical five-factor model, *ESF* exploratory six-factor model, *CFI* comparative fit index, *TLI* Tucker-Lewis index, *SRMR* standard root mean square residual, *RMSEA* root-mean-square error of approximation, *LO90/HI90* lower/upper 90% confidence interval of the RMSEA

### MI across populations

As shown in Table 4, we established configural, weak, and strong MI across the undergraduate and clinical samples. However, the acceptable index criteria were not met for strict MI. We allowed the residual variances of items with the largest modification indices to be freely estimated until the △CFI of the last model was ≤0.01. Parameter constraints of items 15, 4, 14, 18, 17, 20, 7, 12, 24 and 5 were released in this process. Subsequently, partial strict MI of our modified six-factor model was supported. Hence, ultimately, our modified six-factor PID-5-BF model achieved configural MI, weak MI, strong MI, and partial strict MI across our undergraduate and clinical samples.

**Table 4:** Fit indexes of the PID-5-BF for MI across population

Model	S-Bx2	df	CFI	TLI	RMSEA	△CFI	△TLI	BIC	△RMSEA
Configural	3013.479*	420	0.923	0.907	0.040	–	–	323,186.073	–
Weak	3196.737*	437	0.918	0.905	0.041	−0.005	−0.002	323,230.393	0.001
Strong	3480.394*	454	0.910	0.900	0.042	−0.008	−0.005	323,383.756	0.001
Strict	5377.472*	477	0.855	0.846	0.052	−0.055	−0.054	325,314.743	0.010
Part.Strict	3817.237*	467	0.901	0.892	0.043	−0.009	−0.008	323,635.095	0.001

Part.Strict, partial strict invariance by releasing the residuals of items with largest modification indices; the S-Bχ2 = Satorra-Bentler scaled χ^2^; df, degrees of freedom; *TLI* Tucker-Lewis index, *CFI* comparative fit index, *RMSEA* root-mean-square error of approximation, *BIC* Bayesian information criterion

### Reliability

In the undergraduate sample, we obtained a Cronbach’s α of 0.84, a MIC of 0.21 for the PID-5-BF total scale, and domain MICs in the range of 0.29–0.46. Domain sub-scores correlated significantly with total scores (*r* = 0.38–0.80, all *p* < 0.01). With respect to test-retest reliability over a 4-week interval, we obtained a Pearson correlation coefficient of 0.73 for the total scale, with domain correlation coefficients in the range of 0.50–0.67.

For the clinical sample, we obtained a Cronbach’s α of 0.86, a MIC of 0.19 for the total scale, and domain MICs in the range of 0.27–0.58. Similar to our results with the undergraduate sample, we observed significant correlations between the domain sub-scores and the PID-5-BF total score (*r* = 0.26–0.78).

### Criterion validity

Correlation coefficients for each domain were in the range of 0.64–0.86 in the undergraduate student sample and in the range of 0.69–0.91 in the clinical patient sample. As shown in Table 5, the Interpersonal Relationships domain showed the greatest correlation coefficient with the Negative Affect domain in both samples. Finally, as shown in Table 6, PID-5-BF domain scores correlated significantly with the six PDs in Section III of the DSM-5 (schizotypal, antisocial, borderline, narcissistic, avoidant, and obsessive-compulsive).

**Table 5:** Correlations between the PID-5-BF and full-length PID-5

PID-5-BF domains	220-item PID-5 domains				
	NA	De	An	Ps	Di
Normal sample (*N* = 3985)					
NA	**0.64**^**a**^	0.35^a^	0.33^a^	0.40^a^	0.52^a^
IR	**0.58**^**a**^	0.03	0.32^a^	0.26^a^	0.28^a^
De	0.37^a^	**0.86**^**a**^	0.30^a^	0.47^a^	0.43^a^
An	0.37^a^	0.40^a^	**0.75**^**a**^	0.53^a^	0.43^a^
Ps	0.57^a^	0.49^a^	0.46^a^	**0.85**^**a**^	0.54^a^
Di	0.47^a^	0.32^a^	0.27^a^	0.40^a^	**0.82**^**a**^
Clinical sample (*N* = 395)					
NA	0.69^a^	0.31^a^	0.17^a^	0.30^a^	0.53^a^
IR	0.54^a^	−0.05^a^	0.29^a^	0.23^a^	0.26^a^
De	0.40^a^	0.91^a^	0.12^a^	0.43^a^	0.49^a^
An	0.21^a^	0.30^a^	0.73^a^	0.35^a^	0.27^a^
Ps	0.58^a^	0.50^a^	0.37^a^	0.88^a^	0.55^a^
Di	0.51^a^	0.34^a^	0.20^a^	0.44^a^	0.84^a^

*NA* Negative Affect, *IR* Interpersonal Relationships, *De* Detachment, *An* Antagonism, *Ps* Psychoticism, *Di* Disinhibition. Bold presents the highest correlation coefficient in each row

^a^Correlation is significant at the 0.01 level (2-tailed)

**Table 6:** Correlations between PID-5-BF domains and the six DSM-4 PDs retained in Section III of the DSM-5 and measured by PDQ-4 +

PID-5-BF domains	DSM-4 PDs in Section III of the DSM-5					
	STPD	ASPD	BPD	NPD	AVPD	OCPD
Normal sample (*N* = 7155)						
TS	0.35^a^	0.28^a^	0.53^a^	0.39^a^	0.48^a^	0.35^a^
NA	0.20^a^	0.18^a^	**0.43**^**a**^	0.27^a^	**0.33**^**a**^	**0.25**^**a**^
IR	0.13^a^	0.12^a^	0.23^a^	0.29^a^	0.26^a^	0.19^a^
De	**0.24**^**a**^	0.05^a^	0.31^a^	0.17^a^	**0.36**^**a**^	**0.24**^**a**^
An	0.29^a^	**0.26**^**a**^	**0.27**^**a**^	**0.33**^**a**^	0.23^a^	0.21^a^
Ps	**0.39**^**a**^	0.22^a^	0.44^a^	0.34^a^	0.37^a^	0.33^a^
Di	0.13^a^	**0.28**^**a**^	**0.40**^**a**^	0.22^a^	0.32^a^	0.16^a^
Clinical sample (*N* = 231)						
TS	0.44^a^	0.40^a^	0.66^a^	0.28^a^	0.43^a^	0.21^a^
NA	0.24^a^	0.19^a^	**0.43**^**a**^	0.14^a^	**0.35**^**a**^	**0.21**^**a**^
IR	0.11^a^	0.06^a^	0.25^a^	0.30^a^	0.24^a^	0.13^a^
De	**0.22**^**a**^	0.10^a^	0.43^a^	0.02^a^	**0.31**^**a**^	**0.12**^**a**^
An	0.23^a^	**0.36**^**a**^	**0.23**^**a**^	**0.32**^**a**^	0.07^a^	0.06^a^
Ps	**0.46**^**a**^	0.30^a^	0.49^a^	0.24^a^	0.22^a^	0.23^a^
Di	0.20^a^	**0.41**^**a**^	**0.46**^**a**^	0.12^a^	0.34^a^	−0.00^a^

*NA* Negative Affect, *IR* Interpersonal Relationships, *De* Detachment, *An* Antagonism, *Ps* Psychoticism, *Di* Disinhibition, *TS* Total Score, *STPD* schizotypal personality disorder, *ASPD* antisocial personality disorder, *BPD* borderline personality disorder, *NPD* narcissistic personality disorder, *AVPD* avoidant personality disorder, *OCPD*, obsessive-compulsive personality disorder

Bold means suggested domains of each personality disorder in DSM-5;

^a^Correlation is significant at the 0.01 level (2-tailed)

The results of multiple regression analysis were shown in Table 7. VIF values did not suggest any collinearity problem (all< 5) for the PID-5-BF scales. In summary, the PID-5-BF scales predicted a significant amount of variance in the PDQ-4+. Psychoticism was the best predictor in almost all of the six PDs except for the antisocial personality disorder, and the Interpersonal Relationships domain was the second-best predictor of the NPD, AVPD, and OCPD.

**Table 7:** Regression analysis results for PID-5-BF domains’ predictive effect of six SCID-II PDs in undergraduate students

	Overall R^2^	△R^2^	β	VIF
STPD	0.180^a^			
**Ps**		0.150^a^	1.191^a^	1.678
An		0.018^a^	0.653^a^	1.340
Di		0.007^a^	−0.427^a^	1.369
IR		0.004^a^	0.169^a^	1.089
**De**		0.001^a^	0.151^a^	1.516
ASPD	0.128^a^			
**Di**		0.076^a^	0.532^a^	1.369
**An**		0.029^a^	0.549^a^	1.340
De		0.012^a^	−0.459^a^	1.516
Ps		0.011^a^	0.319^a^	1.678
IR		0.001^b^	0.038^b^	1.089
BPD	0.296^a^			
Ps		0.196^a^	0.817^a^	1.669
**NA**		0.067^a^	0.632^a^	1.441
**Di**		0.020^a^	0.539^a^	1.462
IR		0.011^a^	0.272^a^	1.089
De		0.002^a^	0.233^a^	1.488
NPD	0.208^a^			
Ps		0.113^a^	0.766^a^	1.663
IR		0.059^a^	0.511^a^	1.082
**An**		0.032^a^	0.871^a^	1.352
NA		0.004^a^	0.258^a^	1.362
De		0.001^a^	−0.177^a^	1.515
AVPD	0.242^a^			
Ps		0.134^a^	0.480^a^	1.669
IR		0.042^a^	0.479^a^	1.089
**De**		0.047^a^	0.938^a^	1.488
**NA**		0.014^a^	0.343^a^	1.441
Di		0.006^a^	0.365^a^	1.462
OCPD	0.147^a^			
Ps		0.108^a^	0.890^a^	1.735
IR		0.022^a^	0.381^a^	1.102
**NA**		0.009^a^	0.336^a^	1.466
**De**		0.005^a^	0.415^a^	1.535
Di		0.003^a^	−0.289^a^	1.474
An		0.001^b^	0.129^b^	1.363

*NA* Negative Affect, *IR* Interpersonal Relationships, *De* Detachment, *An* Antagonism, *Ps* Psychoticism, *Di* Disinhibition, *STPD* schizotypal personality disorder, *ASPD* antisocial personality disorder, *BPD* borderline personality disorder, *NPD* narcissistic personality disorder, *AVPD* avoidant personality disorder, *OCPD* obsessive-compulsive personality disorder, *VIF* variance inflation factor,△R2 = The change of Model explicable variability after adding the variable; β = regression coefficients

Bold means suggested domains of each personality disorder in DSM-5;

^a^Correlation is significant at the 0.01 level (2-tailed),^b^Correlation is significant at the 0.05 level(2-tailed)

## Discussion

Similar to most previous studies of the PID-5-BF in other countries [14–16, 19, 20], the theoretical five-factor model was acceptable, but an exploratory six-factor model was more suitable and culturally-adapted in our Chinese sample. Following modifications, the Chinese six-factor model of PID-5-BF achieved configural MI, weak MI, strong MI, and partial strict MI across undergraduate and clinical samples. In agreement with prior studies [14, 20], we found that the PID-5-BF was highly correlated with the original 220-item PID-5, and the domains generally correlated with and predicted the six PDs retained in Section III of the DSM-5. We obtained acceptable Cronbach’s α and MIC values for the PID-5-BF [54], revealing a good internal consistency of PID-5-BF, similar to prior studies [16, 17, 20]. Furthermore, our finding of good test-retest reliability over a 4-week interval is in agreement with prior work showing similarly good test-retest reliability of the PID-5-BF over a 2-week interval in a sample of high school students [16].

### Factor structure

Both the theoretical five-factor model and exploratory six-factor model were acceptable, while our results demonstrated that the six-factor structure was more culturally informed. Interestingly, a prior study conducted with Filipino college students also showed a relatively poor fit of the theoretical five-factor model for the PID-5-BF [22]. Thus, we speculate that the discrepancy may reflect differences in the way people from Western versus Eastern cultures understand personality constructs and thus interpret items of the PID-5-BF.

Four of the factors in our exploratory six-factor model were consistent with the theoretical five-factor structure. Only the Negative Affect domain failed to align, and items 10 (fear being alone) and 19 (I crave attention) were placed in the newly added factor called Interpersonal Relationships. According to traditional personality theories in Western cultures, which focus on internal characteristics of the individual [57], item 10 may remind people of loneliness, which is considered as the experience of negative feelings about missing or lacking relationships, emphasis of which is on emotion. On the contrary, in Eastern cultures, which are generally more collectivist, individuals are often considered to be inherently closely connected with others [58]. Accordingly, being alone may be viewed as social isolation, referring to the absence of relationships with others [59]. In Chinese culture, item 10 may express one kind of fear of being socially isolated, which failed to achieve interpersonal harmony. Item 19, which is associated with the Antagonism domain of the original PID-5-BF, refers to behaviors that put the individual at odds with others, including an exaggerated sense of self-importance and an expectation of special treatment [10]. However, in a collectivist society, one’s sense of belonging is an important aspect of his or her personality constitution [60]. Consequently, item 19 is likely to be understood as one’s desire to fit into a certain group and to communicate with others. In the Chines culture, item 19 is correlated with Face, the concept of which is more interpersonally connected [32]. Being focused on by others could increase one’s face and enhance confidence in social settings. Prior studies have explored the influences of collectivist versus individualist cultures on personality [60–62]. In summary, cultural differences in how one understands and interprets Items 10 and 19 may affect the factor loading of these two items.

The unique structure of the DSM-5 personality trait model in Chinese respondents, compared to respondents from most other examined countries, may be related to cultural differences in general personality models [43, 63]. Although the five-factor model has been widely used globally, it may not be fully applicable in a variety of cultural contexts due to its Western-central derivation. Consistent with this supposition, the five-factor model was not well-fitted when the NEO Personality Inventory was examined in the Philippines [24], Korea [25], and Japan [26]. Hence, it appears that the Big-Five Model does not fully explain personality traits in collectivist society contexts [36]. Prior studies have proposed a six-factor hypothesis of Chinese personality traits, with the addition of Interpersonal Relationships [28]. The importance of this sixth dimension for Chinese personality analysis has been affirmed in the Chinese Personality Assessment Inventory (CPAI) standardization studies [37], which contributed to the Big Six personality model. A recent study examining the relationships between Big Six personality and entrepreneurial intention discovered the decisive effect of the Interpersonal Relationship dimension in the Chinese settings for the first time [35], which was a shred of strong evidence to support the standpoint that the five-factor model was acceptable while the six-factor structure had more cultural applicability in East-Asian contexts. Moreover, this cultural-specific factor in PID-5-BF would help us to comprehend personality disorders from a cultural perspective.

### MI

To the best of our knowledge, this study is the first to explore MI of the PID-5-BF. The establishment of MI provides evidence of a consistent underlying structure across groups and thus enables group means to be compared [38]. When performing nested MI modeling, as was done here with configural, weak, strong, and strict MI, MI must be established sequentially from lower- to higher-level MI analyses [64]. We were able to achieve MI fully with respect to factor structure (configural MI), metric (weak MI), and intercept (strong MI) equivalences for the PID-5-BF in both samples. Strict invariance was partially satisfied.

Because our modification index analyses led us to release constraints on Items 15, 4, 14, 18, 17, 20, 7, 12, 24, and 5 to better achieve strict invariance, it can be deduced that the residual variances of these items were not equivalent across our two sample groups. Notwithstanding, upon achieving strong MI, we were able to conclude that our finding of higher PID-5-BF scores in our clinical sample, compared to our undergraduate sample, could be considered a reliable finding. Moreover, these data affirm a satisfactory discriminant validity of the PID-5-BF for differentiating between nonclinical and clinical individuals.

### External validity and clinical value

Although the 220-item PID-5 has many merits for personality diagnosis—such as close relations with clinical symptoms, the ability to be combined with various psychotherapy methods, and good stability over time [12]—its length hinders its clinical utility. Pires et al. (2018) examined the psychometric properties of the 220-item (original), 100-item (short form), and 25-item (brief form) PID-5 versions in a sample of Portuguese university students and concluded that any of the three could be used to assess maladaptive personality traits reliably and validly [18]. Bach et al. (2016) compared the three forms in a Danish population and showed that the three scales were highly similar with respect to internal consistency, factor structure, discriminant validity, and correlation with DSM-4 PD dimensions [14]. The present findings of very strong correlation coefficients between the PID-5-BF and the 220-item PID-5 in both of our samples indicated that in addition to saving time, reducing the burden upon participants, and being generally more clinic friendly, the PID-5-BF maintains the validity of the original instrument to a remarkable degree.

Moreover, the factors of the PID-5-BF in our six-factor model showed good alignment with the six PDs in Section III of the DSM-5. Similar to a previous study [20], the Psychoticism domain correlated highly with all of the PDs in the undergraduate sample and we speculated that as items in the Psychoticism domain were much different from daily life, most college students would be sensitive to abnormal behaviors, which thereby limiting and attenuating the specificity of Psychoticism. All domains had significant relations with one or more PDs, which was consistent with the previous study [14], and each PD was significantly associated with expected domains of PID-5-BF. However, the discriminant validity was not impressive, which may due to the range restriction of the sample. The significant but unspecific correlation between PID-5-BF and PDQ-4+ embodied the consistency and diversity between DSM-5 and DSM-4. Furthermore, it may reflect the trend from categorical scheme to dimensional frame in PD diagnosis, which reminders us that PID-5- traits might all relate to a general factor of maladaptivity [3, 14].

Multiple regression analysis revealed that the Interpersonal Relationships domain was the second-best predictor of the NPD, AVPD, and OCPD, which could make sense in theory. For example, NPDs always attempt at regulation through attention and approval seeking from others, relationships are largely superficial. AVPDs avoid social situations and inhibit interpersonal relationships. OCPDs have difficulties in establishing and sustaining close relationships [10]. All of the three PDs have a high proportion of maladjustment in interpersonal relationships. The reason why the IR factor correlated weakly with BPD may be that IR was more closely related to interpersonal sensitivity and withdrawal but BPD was relevant to a long-standing pattern of instability in interpersonal relationships. Except for the Psychoticism domain, nearly every PD’s sorting of predictors was conformed to the theoretical hypothesis or clinical observation. In summary, the PID-5-BF retained satisfactory psychometric properties, despite its extensive omission of items relative to the 220-item PID-5, affirming its suitability as a preliminary clinical PD screening tool.

The PID-5-BF can be used to differentiate between psychologically healthy and troubled respondents, at least preliminarily. Bach et al. (2016) reported that the PID-5-BF has very good discriminant validity between psychiatric outpatients and community-dwelling individuals [14], consistent with our findings of significantly higher PID-5-BF scores in our clinical patients than in our undergraduate students’ sample. Clinicians can administer the PID-5-BF to acquire a rough estimation of one’s personality functioning, laying the foundation of further treatment planning, and then judge the need for additional assessments. Although the PID-5-BF may not provide unique clinical information regarding specific symptoms, it can describe personality traits through the assessment of dimensions, embodying differences in degree rather than in a category, contributing to individualized therapy development.

### Limitations and future directions

The present study had some noteworthy limitations. First, the retested sample and clinical population were relatively small due to practical limitations. Second, the clinical sample was heterogeneous, including patients diagnosed with various psychological disorders. Third, the current study was cross-sectional, and cross-sectional studies cannot demonstrate predictive validity with the robustness of longitudinal studies. Hence, there is a need for larger longitudinal and clinical-sample studies of the PID-5-BF, particularly with samples constituted by patients with PDs.

Finally, the current study explored a novel six-factor model for the PID-5-BF in Chinese samples, the validity of which should be tested in 220-item PID-5 in further studies. This Chinese six-factor model was supported by previous studies on personality traits in Chinese samples [36, 37]. However, the six-factor structure limited the possibility to compare with or generalize to some advanced researches using the original PID-5-BF operationalization. For instance, recently the combination of DSM-5 and ICD-11 in the diagnosis of personality disorders has aroused increasing attention [65–71]. Researchers developed some novel algorithms to assess ICD-11 personality domains from the five-factor structure and relevant sub-scores of PID-5 [70, 71]. It’s worth noting that in 2018 Oltmanns et al. developed a new instrument named the personality inventory for ICD-11(piCD) to assess five trait dimensions of ICD-11 [72], which indicated that combination of PID-5-BF and piCD for personality disorder study would become a more direct and reasonable way to explore the relationship between these two diagnostic systems in the Chinese sample.

On the other hand, because dimensions represent continua from normal to abnormal, actionable score ranges need to be established based on ample empirical data collected in clinical practice rather than developed from theoretical hypotheses. Further correlational analyses between the PID-5-BF and other psychological scales are also needed. MI of the PID-5-BF should also be further examined across genders, age bands, and cultures, particularly in Asia and the Pacific Islands.

## Conclusion

Psychometric properties of the Chinese version of PID-5-BF were partially supported as the factor of Negative Affect was not replicated in its entirety. It was divided into two domains and we named the novel domain Interpersonal Relationships. Based on the current study, both the theoretical five-factor model and the exploratory six-factor model of PID-5-BF were acceptable for the Chinese population. The five-factor model allows for comparison and integration with other work on the original theoretical model. However, the Chinese six-factor structure may be more culturally informed in East Asian settings. In general, the PID-5-BF is suitable for assessing personality traits and clinical screening for PDs quickly.

## Data Availability

The datasets generated and analyzed during the current study are not publicly available due to no permission from participants to share anonymized participant data publicly but are available from the corresponding author on reasonable request.

## Abbreviations

PID-5-BF: Personality Inventory for DSM-5 Brief Form
PD: Personality disorder
EFA: Exploratory factor analysis
CFA: Confirmatory factor analysis
MI: Measurement invariance
PDQ-4 +: Personality diagnostic questionnaire-4+
